# Prediction of P300 BCI Aptitude in Severe Motor Impairment

**DOI:** 10.1371/journal.pone.0076148

**Published:** 2013-10-18

**Authors:** Sebastian Halder, Carolin Anne Ruf, Adrian Furdea, Emanuele Pasqualotto, Daniele De Massari, Linda van der Heiden, Martin Bogdan, Wolfgang Rosenstiel, Niels Birbaumer, Andrea Kübler, Tamara Matuz

**Affiliations:** 1 Institute of Psychology, University of Würzburg, Würzburg, Germany; 2 Institute of Medical Psychology and Behavioral Neurobiology, University of Tübingen, Tübingen, Germany; 3 Department of Computer Engineering, University of Tübingen, Tübingen, Germany; 4 Psychological Sciences Research Institute, Université catholique de Louvain, Louvain-la-neuve, Belgium; 5 Ospedale San Camillo, Istituto Di Ricovero e Cura a Carattere Scientifico Fondazione, Venezia-Lido, Italy; 6 Computer Engineering, University of Leipzig, Leipzig, Germany; 7 Graduate Training Centre of Neuroscience, International Max Planck Research School, Tübingen, Germany; 8 Department of Cognitive Psychology, University of Finance and Management, Warsaw, Poland; University of Modena and Reggio Emilia, Italy

## Abstract

Brain-computer interfaces (BCIs) provide a non-muscular communication channel for persons with severe motor impairments. Previous studies have shown that the aptitude with which a BCI can be controlled varies from person to person. A reliable predictor of performance could facilitate selection of a suitable BCI paradigm. Eleven severely motor impaired participants performed three sessions of a P300 BCI web browsing task. Before each session auditory oddball data were collected to predict the BCI aptitude of the participants exhibited in the current session. We found a strong relationship of early positive and negative potentials around 200 ms (elicited with the auditory oddball task) with performance. The amplitude of the P2 (*r*  =  −0.77) and of the N2 (*r*  =  −0.86) had the strongest correlations. Aptitude prediction using an auditory oddball was successful. The finding that the N2 amplitude is a stronger predictor of performance than P3 amplitude was reproduced after initially showing this effect with a healthy sample of BCI users. This will reduce strain on the end-users by minimizing the time needed to find suitable paradigms and inspire new approaches to improve performance.

## Introduction

One of the earliest discussions of a communication channel that is independent of muscular control can be found in Vidal et al. [1]. These communication systems, termed brain-computer interfaces (BCIs), use components extracted from the electroencephalogram (EEG) as a control signal. Control signals include slow cortical potentials (SCPs), the P300 event-related potential (ERP) component, visually evoked potentials (VEPs) and the sensorimotor-rhythm (SMR) [2], [3], [4], [5]. Brain activity used for BCI control can also be measured with functional magnetic resonance imaging (fMRI) [6], [7], [8], electrocorticography (ECoG) [9], [10], magnetoencephalography (MEG) [11] and functional near infared red spectroscopy (fNIRS) [12]. Recent studies have also shown that the EEG response to yes and no questions can be conditioned and may be used to control a binary communication system [13], [14], [15], [16]. Hybrid-BCIs combine different control signals [17], as extensively reviewed by van Gerven et al. [18].

The P300 BCI paradigm was first introduced by Farwell and Donchin [3]. The user shifts his or her attention to a single letter in a matrix. The rows and columns of this matrix then flash in a random pattern. When the row or column containing the letter the user is focusing on flashes this elicits a P300. The P300 following the target stimulations is larger than the P300 following non-target stimulations which enables the classification of the intended row and column. Classification requires repetition of flashes. Averaging trials decreases the amplitude of the spontaneous EEG and conserves the amplitude of the stimulus-locked P300. This increases the signal-to-noise ratio (SNR) enabling successful classification. Implementations of this BCI paradigm commonly use the linear discriminant analysis LDA algorithm (and derivations thereof) for classification [3], [19], [20], [21]. For a review of classification algorithms for BCIs see Lotte et al. [22].

Persons with severe motor impairments are differentiated into the LIS and the complete locked-in state CLIS [23]. Persons in the LIS retain residual muscle control for basic communication. Persons in the CLIS have no muscle control and cannot communicate. Before entering the CLIS BCI performance is not influenced by the severity of the disease [24]. To date attempts to achieve this goal have failed [23], [25].Therefore, it is one of the primary goals of BCI research to restore communication for persons in the CLIS. In amyotrophic lateral sclerosis (ALS) the transition from LIS to CLIS is gradual and the precise time point is hard to define. In a single case study, a person with ALS lost control of facial muscles and the external anal sphincter before losing control of eye movements [25]. Besides ALS, other disorders can lead to LIS or even CLIS. These include brainstem stroke, cerebral palsy, muscular dystrophies and spinal cord injury (SCI). As communication is a basic need, BCI could potentially contribute to maintain or regain or even improve quality of life [26]. Recently, it has been proposed to use BCIs as an alternative method of cognitive assessment in persons with ALS [27]. Reliable cognitive assessment will improve the way BCIs can be tailored to the needs of the individual.

Healthy users achieved average accuracies above 90% using visual P300 BCI spellers [28]. In a sample of *N*  =  81 healthy participants eleven percent did not reach accuracies above 80% [29]. In samples of motor impaired persons the accuracies achieved with BCIs often decrease dramatically. In a study with four motor impaired participants, two did not achieve accuracies above 70%, in the second phase one of four [30]. A study by Kübler and Birbaumer showed data from eleven persons with motor impairments (in LIS) that achieved an average accuracy of 66% [23]. Of these eleven, four achieved a level of control considered sufficient for independent use of BCIs (which was not defined as a quantitative but a qualitative measure evaluating the person’s ability to use a BCI for tasks such as communication, web surfing or environmental control). Two more reached accuracies above 70% correct symbol selection, the criterion threshold at which the use of communication aids becomes feasible [31], [32]. Thus in this sample, 45% of the participants were unable to freely communicate with a visual P300 BCI.

Several studies have described physiological and psychological predictors of BCI performance. Hammer et al. showed that the ability to concentrate and visuo-motor coordination predict SMR BCI performance [33]. More recently motor imagery questionnaires have been shown to be a strong predictor of BCI performance [34]. From a physiological perspective, Blankertz et al. have shown that the amplitude of the resting SMR peak correlates strongly with subsequent SMR BCI performance [35]. Using fMRI, Halde et al. were able to demonstrate that SMR BCI users with high aptitude and low aptitude have identical neural activity elicited by motor execution but differ during motor imagery and particularly motor observation [36]. During motor observation the number of activated voxels correlated significantly with BCI-performance (*r*  =  0.53). Specifically, the number of activated voxels in the right middle frontal gyrus was correlated with BCI-performance (*r*  =  0.72). This underlines the importance of task monitoring and working memory throughout the BCI session. Structural differences between high and low aptitude users were also shown [37]. A volumetric analysis showed no differences between the groups but structural integrity and myelination quality of deep white matter structures were strong predictors of BCI-performance. Studies with users of SCP BCIs have shown the predictive power of performance in early sessions for later performance [38]. More specifically, the number of sessions needed to achieve significant cursor control correlated moderately with the number of sessions required to achieve criterion level control (above 70%, [39]).

Concerning P300 BCIs, motivation impacts the performance achieved in a subsequent BCI session [28]. The authors described a reduced P300 amplitude for the least motivated participants as opposed to the most motivated participants. A moderate but significant correlation between spectral power in the high alpha and the low beta band during a baseline recording and subsequent P300 BCI performance has also been shown [40]. The data originated from measurements performed over the course of two years with one person with ALS. The authors analyzed a total of 197 runs in which the participant spelled 1200 letters. Using resting state data recorded in a different session than the P300 BCI test sessions, it has been shown that the frequency band of the maximum peak in the power spectrum correlates strongly with visual P300 BCI performance [41]. Additionally, spectral and temporal EEG features correlate with performance during BCI usage [42].

Physiological factors not extracted from the EEG can also influence BCI performance. In a study performed with healthy participants the relationship between heart rate variability and P300 BCI performance was demonstrated [43]. In another study it was shown that an auditory oddball recorded before the P300 BCI session can be used to predict performance in a sample of 40 healthy participants [44]. It has also been demonstrated in one person in the CLIS and two in the LIS that oddball data can be used to predict performance in a classical conditioning BCI [16]. An overview of existing predictors can be found in Table 1. The predictors are grouped according to the categories of the model suggested by Kübler [45].

**Table 1:** Overview of existing predictors.

Predictor	Category	Paradigm	Publication
Initial performance	Psychological	SCP	[38]
Locus of control	Psychological	SMR	[87]
Concentration/Coordination	Psychological	SMR	[33]
Motivation	Psychological	Visual P300	[28]
Motor imagery questionnaires	Psychological	SMR	[34]
Resting alpha/beta peak	Physiological	Visual P300	[40]
Resting SMR peak	Physiological	SMR	[35]
fMRI	Physiological	SMR	[36]
HRV	Physiological	Visual P300	[43
Resting alpha frequency	Physiological	Visual P300	[41]
ERP+power spectrum	Physiological	Visual P300	[42]
Oddball ERP	Physiological	Visual+AuditoryP300	[44]
DTI	Anatomical	SMR	[37]

We give the name of the variable used for prediction of performance, the category out of psychological, physiological and anatomical (this category was used to group the entries of the table), the BCI paradigm that was used to determine performance and a reference to the corresponding publication.

In this paper we propose using an auditory oddball session performed before the P300 BCI session to predict performance. Instead of a sample of healthy participants (see [44]) we apply this method of performance prediction to a sample of severely motor impaired persons. We chose to use an auditory oddball instead of a visual oddball to have a predictor that is independent of the visual system, which becomes compromised e.g. in later stages of ALS. Due to a stronger dependence of the ERP morphology on stimulus discriminability, intensity and probability we do not assume that using the auditory instead of the visual modality will be a disadvantage [46], [47], [48], [49,]. Using a short BCI session instead of the oddball for prediction requires that all instructions are understood by the user in the first session. Any misunderstandings will introduce an unwanted bias in the prediction. Performing the auditory oddball requires little attentional resources and the instructions are easy to understand. For this reason, an auditory oddball as we used in this study has also been used to assess brain function in severly brain injured participants [50]. Thus, we conclude that the auditory oddball represents a robust and generally applicable solution for testing in a sample of severly motor impaired BCI users. We evaluated the success of our method by analyzing which components of the P300 ERP elicited by the auditory oddball task correlated with later BCI performance.

## Methods

### Participants

Eleven persons with motor impairments (6 male, mean age 54.36 years, standard deviation (*SD*) 10.89 years, range 36–71 years) participated in three sessions of the P300 BCI performance prediction study (see Table 2). We selected participants based on their willingness to participate and general suitability for EEG studies (no skin irritations, epilepsy) and recruited them through local support groups. There was no financial compensation and all participants were informed that the system could not be provided to them for personal use after the study. The main motivation of participation is to contribute to the development of BCI systems so that others may profit in the future. The level of impairment ranged from zero to 43 (mean 17.7) according to the ALS functional rating scale revised (ALS FRS-R) [51]. This instrument evaluates the ability of the participant to carry out activities of daily living such as speech and handwriting. Lower values indicate a lower level of functionality. The ALS FRS-R questionnaire evaluates twelve items with a score between zero and four. Thus the score can range from zero (maximally disabled) to 48 (not disabled). We informed each participant about the purpose of the study and each participant gave informed consent prior to the experimental session. The participants gave informed consent in the presence of their legal representative. The consent was given using residual movements that had been agreed on before. Written consent was then provided by the legal representatives (if the participants were unable to provide written consent).The Ethical Review Board of the Medical Faculty of the University of Tübingen approved the study and consent procedure.

**Table 2:** Description of participants with motor impairment.

Participant	Sex	Age (years)	Year of diagnosis	ALS diagnosis	ALS FRS-R	Artificial nutrition	Ventilation
1	Male	71	2005	Spinal	11	No	Yes (non-invasive)
2	Male	54	2006	Spinal	23	No	No
3	Male	70	2008	Spinal	18	No	Yes (non-invasive)
4	Female	50	2003	Bulbar	17	No	Yes (non-invasive)
5	Female	53	2008	Spinal	23	No	No
6	Male	36	1976 (Duchenne)	N/A	7	No	Yes (non-invasive)
7	Male	55	2003	Spinal	43	No	No
8	Female	48	2007	Spinal	12	No	Yes (non-invasive)
9	Male	65	2009	Bulbar	34	No	No
10	Female	42	1996	Spinal	7	No	No
11	Female	54	2005	Spinal	0	Yes	Yes (invasive)

Participant six has Duchenne muscular dystrophy. Gender, age in years, year of diagnosis, ALS onset (bulbar or spinal) as well as the score of the ALS functional rating scale at the time of the study.

### Experimental Design

**Figure 1:**
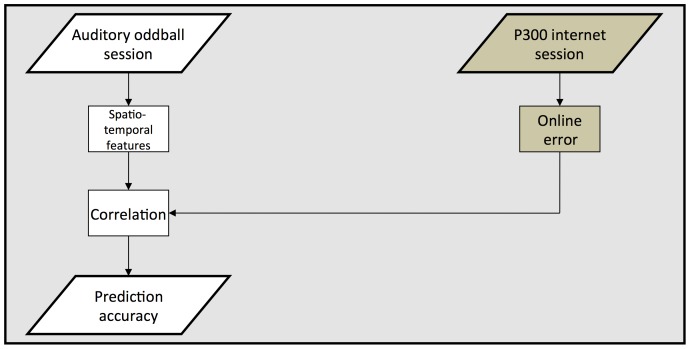
In each session every participant performed an auditory oddball and a visual P300 BCI internet browsing task. The participants performed three sessions in total. We averaged the results of the web browsing task across the three sessions to determine the performance of each participant. The BCI system provided online feedback. We evaluated performance using the number of errors made by the participant. Features extracted from the auditory oddball session served as a performance measure and we assessed whether we can use them to predict BCI aptitude of participants with severe motor impairment in the visual P300 BCI task.

The participants of this study took part in two separate experiments. First, an auditory standard oddball experiment, to predict the performance in the second, a visual P300 BCI task. We provide an overview of the design in Figure 1.

### Auditory Oddball

The auditory stimuli were comprised of a set of standard tones (duration 160 ms; chords at 517 Hz, 646 Hz and 775 Hz) and deviants (the oddball; duration 160 ms; a 517 Hz tone). The ratio of standards and deviants was 4∶1. Each sequence of stimuli consisted of five tones. One run consisted of 20 sequences. In total we performed three runs resulting in 60 deviant and 240 standard tones (one session). We instructed the participants to count the deviants. The inter stimulus interval (ISI) was 800 ms, resulting in a run length of 96 s and a total length of the experiment of 288 s (4 min 48 s).

### The Visual P300 BCI

**Figure 2:**
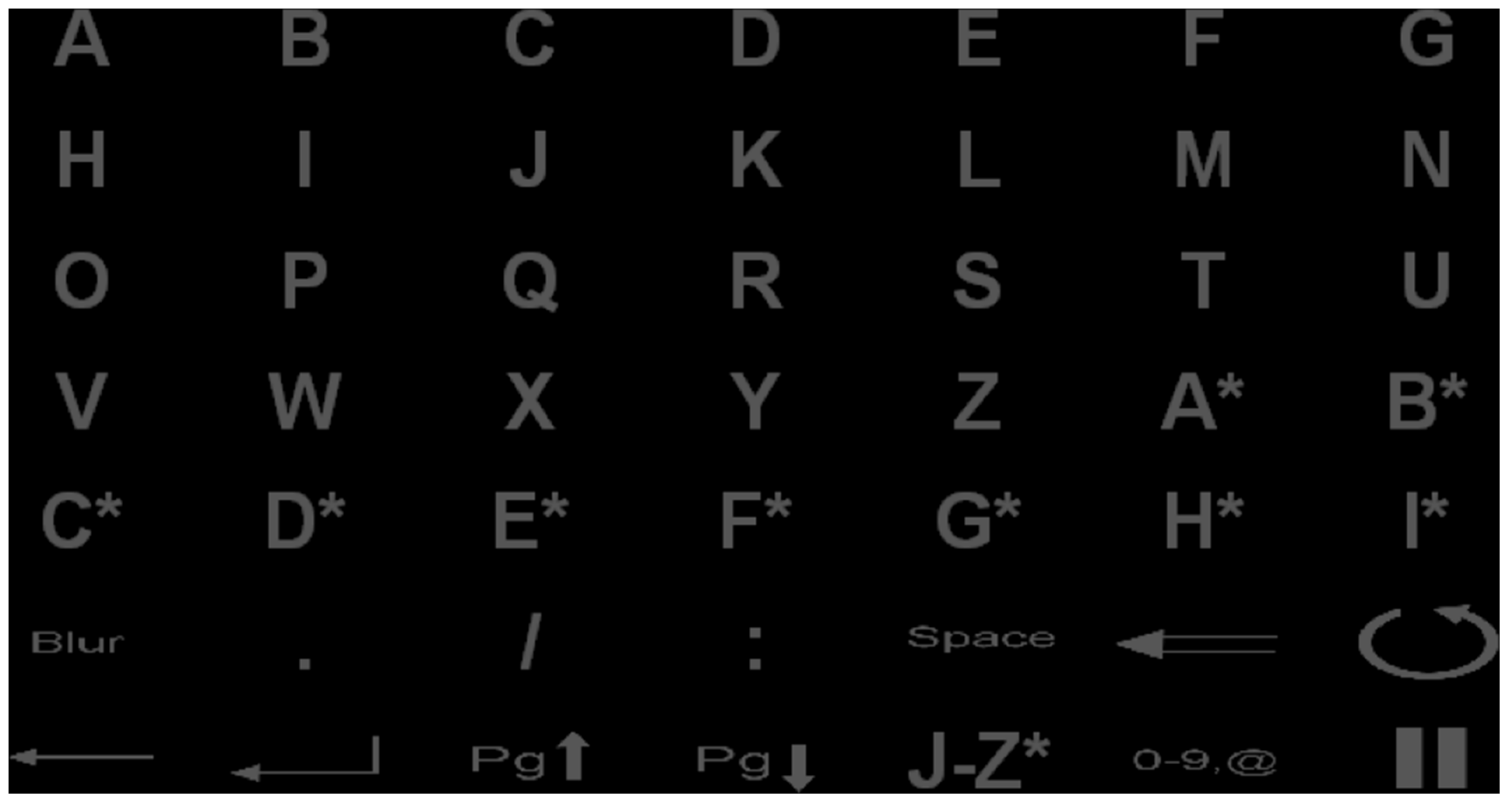
The visual P300 BCI matrix the participants used to control the web browser. Participants used letters to select hyperlinks and for text input. The participant selected links on websites with more than 26 links using the letters marked with a “*”. Other functions included moving forward and backward between pages or reloading. The system provided submatrices for numbers and double letters from J–Z*.

During the visual P300 BCI experiment participants attended a nested matrix designed to control a web browser [52]. We included the initial matrix as depicted in Figure 2. We will present the detailed results of the web browsing task independently of the performance prediction study and spared the participants the strain of additional conventional P300 speller sessions that would otherwise have been needed. Thus, we used the P300 BCI web browsing sessions for performance prediction. The matrix had an initial dimension of 7×7 (see Figure 2) from which the user can navigate to two different 5×6 matrices (bottom row, “J–Z*” and “0–9,@”). One sequence therefore comprised between 11 (5×6 matrix) and 14 (7×7 matrix) flashes (one for each row and column) of 62.5 ms duration followed by a 125 ms inter-flash interval. We set the number of flashes to a subject specific level based on a preceding measurement with a conventional 6×6 speller matrix. During the training measurement the participants spelled 17 letters without feedback in two separate runs. Each stimulus was presented 15 times. We used these two runs to train the classifier for online feedback with the visual P300 BCI. All participants performed a third run spelling eleven letters with feedback to verify the selected settings.

During the web browsing task, the system paused for 8 s after symbol selection in which it performed signal classification and presented the selected letter to the participant. We chose the length of the pause to give the next website enough time to load and the user enough time to select the next action. The participants had to perform a given sequence of tasks using the web browser and make a minimum of 40 correct selections before the tasks were completed. The number of selections needed to complete the task successfully varied between users. We used the number of errors instead of more conventional measures such as accuracy or bitrate because different errors may need different numbers of selections to correct (depending on what command the participant sent to the web browser erroneously). Thus, making a mistake and then needing to perform correcting steps may actually increase accuracy in retrospect. An increase in accuracy occurs if the error needs multiple correct selections to be undone. This can e.g. occur if the user hits enter by mistake when entering a term in a search field. To correct it the user must select the “backwards” function, reselect the search field and complete, correct or possibly re-enter the search term. As a consequence these multiple (ideally) correct selections will increase accuracy even though the total time until the intended goal is completed has also increased. Thus, in our scenario performance (minimum time needed for achieving the pre-defined goal) would have decreased. Then again it is not the aptitude of the user that increases the number of corrective steps needed after a particular mistake which makes using the total time needed for that task another suboptimal measure. This is the reason for choosing the absolute number of errors made by the participant. Each of the eleven participants performed three sessions using the P300 web browser. For all comparisons between low and high aptitude users we split the group at the median. For the correlation analysis, the number of errors was averaged across sessions for each participant.

### Data Acquisition

We performed stimulus presentation and data collection with the BCI2000 software [53]. We recorded the EEG using an Ag/AgCl electrode cap with 16 channels (manufactured by EASYCAP GmbH, Herrsching, Germany; F3, Fz, F4, T7, T8, C3, Cz, C4, Cp3, Cp4, P3, Pz, P4, Po7, Po8 and Oz) based on the modified 10–20 system of the American Electroencephalographic Society [54]. The reference was placed on the right and the ground on the left mastoid. The sampling rate was set to 256 Hz with a high pass filter at 0.1 Hz and a low pass filter at 60 Hz (auditory oddball: 30 Hz) using a g.tec 16-channel gUSBamp EEG amplifier (g.tec medical engineering GmbH, Austria).

### Offline Processing

During the offline processing we high-pass filtered the data acquired during presentation of the auditory oddball at 0.5 Hz and then low-pass filtered at 20 Hz using a two-way least-squares FIR filtering by a function from the EEGLAB toolbox [55].

To isolate and remove ocular artifacts we employed the blind source separation (BSS) method algorithm for multiple unknown signals extraction (AMUSE) [56], [57]. AMUSE is particularly suited to remove ocular artifacts [58]. To increase external validity we performed no other artifact correction or rejection.

For offline analysis we replaced the right mastoid reference with a common average reference (CAR). The CAR re-references the potential at each electrode with mastoid reference by subtracting the average potential of all electrodes. After segmenting the data into individual epochs (0–800 ms), we baseline corrected by subtracting from every epoch the mean amplitudes in the −100 to 0 ms pre-stimulus interval.

We defined amplitude of the P300 as the local maximum between 250 and 700 ms, the N1 as the local minimum between 100 and 200 ms, the P2 as the local maximum between 200 and 250 ms and the N2 as the local minimum between 250 and 375 ms. Note that using the CAR also influences the topography of the investigated ERP components. For example components which have a frontal maximum absolute value will appear to have a dipolar topography after applying the CAR. Negative components on Fz will appear positive on Oz if the negative amplitude was high enough. Thus we inverted the sign for the analysis of the ERP peaks for electrodes posterior to Cz. This effect must also be taken into consideration when evaluating the topography of the ERPs.

### Classification

We used stepwise linear discriminant analysis (SWLDA) for online and offline classification. This algorithm is commonly employed as a classification method for visual and auditory P300 BCIs [3], [30], [59], [60], [61]. The algorithm adds the most significant features to the model first (*p*<0.1, otherwise the model generation fails). Then the algorithm adds the remaining features to the model in order of their significance. During the backward stepwise regression step the algorithm removes features from the model that do not fulfill a significance level of *p*<0.15. After 60 iterations or if no further features fulfill the inclusion criterion the model generation stops and the model is applied to the data in the current state.

We smoothed the spatiotemporal features of each trial with a moving average filter, with a width of 25 samples, and then decimated them by a factor of 25 prior to feature selection and classification. For classification we used a time window from 0 to 1000 ms and the full channel set for online feedback.

During online classification we applied the model separately to the trials following row and column flashes. The BCI system displays the row and and the column trials with a maximum score as feedback. This means that the system requires no bias term for classification of the P300 responses. The classifier weights were trained on and applied to single trials for feedback. The classifier output was summed over all trials of a particular stimulus and the row and column with the maximum output selected for feedback.

### Statistical Analysis

All *r*-values were calculated as rank correlations which were performed according to the method described in [62]. For the correlation analysis performance of the participants was normalized to the mean performance across the three sessions (to ensure that all values were independent). Significance was assumed for *p*<0.05 for all statistical tests (including t-tests). All results of the ERP analysis were given as mean with standard deviation.

## Results

### Online Performance

Due to the nature of the task (the participants had to accomplish certain goals with a P300 controlled web browser using predefined steps) we give performance in number of errors. The participants made a mean amount of 15.1±13.3 errors (range 0–51 errors) in a task that needed a minimum of 40 selections. We found no correlation between ALS FRS-R and the average error made by each user in the three measurement sessions (*r*  =  0.29, *p*  =  0.39).

### Group Differences in the Auditory Standard Oddball

**Figure 3:**
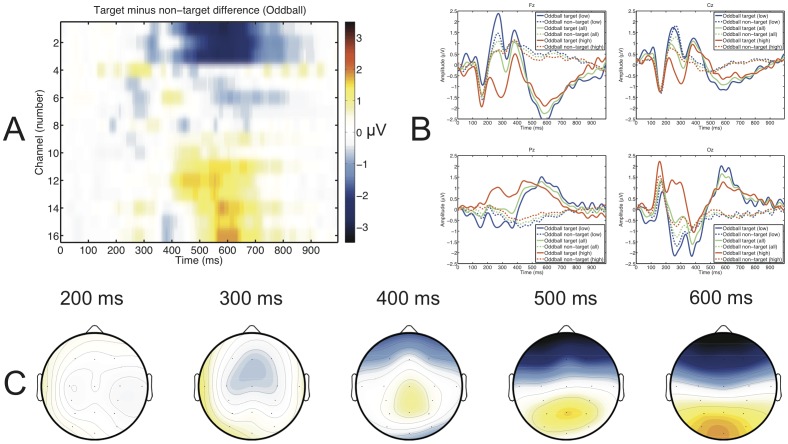
The average amplitude across all participants of the response to the auditory oddball is shown as the full spatio-temporal feature matrix (EEG channels are in the sequence given in the data acquisition section) of the target non-target difference (A). Time course at Fz, Cz, Pz and Oz of the averaged ERP are shown for high aptitude (red), low aptitude (blue) and all users (green) for targets (continuous lines) and non-targets (dashed lines; B). Topographic distribution (the scale is the same as in A) of the target non-target difference at 200, 300, 400, 500 and 600 ms (C).

Target and standard trials of the auditory oddball were evaluated separately for high and low aptitude users. Figure 3 shows the target/non-target differences of the whole spatio-temporal feature matrix in (A). In Figure 3 (B) we show the differences between the ERPs of high (red) and low (blue) aptitude users on electrodes Fz, Cz, Pz and Oz. For comparison purposes, we also plotted the average response across all participants (green). Finally, the topographies of the target/non-target differences from 200 to 600 ms are shown in 100 ms steps.

The averaged auditory oddball P300 ERP component peaks had an amplitude of 2.08±1.3*μ*V at 361.51±87.1 ms on electrode Cz. High aptitude users had an average amplitude of 1.52±1.3*μ*V and low aptitude users of 2.54±1.3*μ*V. An independent t-test showed that the difference was not significant (*t*_9_  =  −1.3, *p*  =  0.23). Latencies averaged to 392.19±91.9 ms for high aptitude and to 335.94±81.7 ms for low aptitude users. A independent t-test was not significant (*t*_9_  =  1.08, *p*  =  0.31).

### Correlation between Auditory Oddball Response and Aptitude

**Figure 4:**
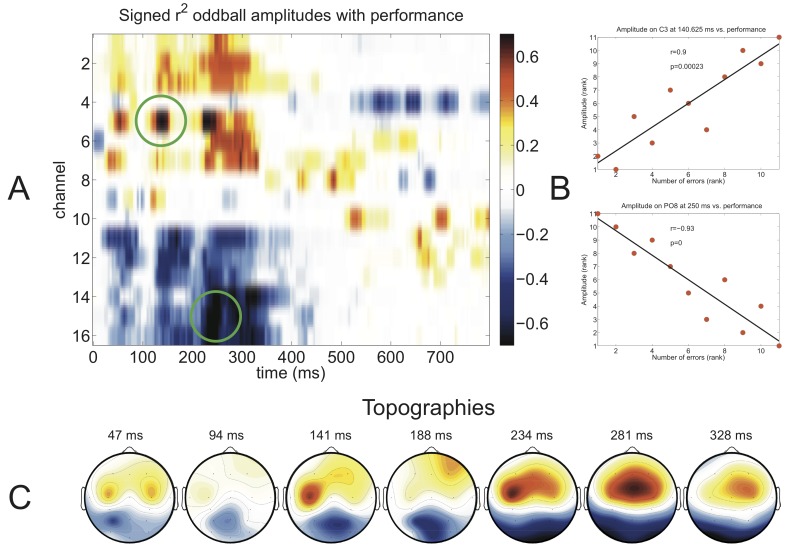
Signed *r* ^2^ values between auditory oddball amplitudes at all time points and channels with visual P300 BCI performance (defined as the number errors during the web browsing task) in red for positive correlations and in blue for negative correlations (A). Two elements were selected from the matrix (marked by green circles in A) for visualization using scatter plots (B) showing a correlation of *r*  =  0.9 (*p* < 0.01) on electrode C3 and a correlation of *r*  =  −0.9 (*p* < 0.01) on electrode Oz. Finally, topographic distributions of the signed *r* ^2^ values are shown at the bottom (C). Note that due to the use of “number of errors” as performance measure positive correlations indicate a decrease in performance with increasing amplitude, whereas negative correlations indicate an increase of performance with decreasing amplitude.

Figure 3 shows that the amplitude differences of later ERP components between the aptitude groups were smaller than the amplitude differences of earlier positive and negative ERP components. This was confirmed by the correlation plot of the spatio-temporal feature matrix shown in Figure 4 (A). The highest *r* ^2^ values were in the latency range from 100 to 350 ms. In Figure 4 (B) the upper of the two scatter plots illustrates the positive correlation between errors in the browsing task and amplitude of the auditory oddball P300 on frontal electrodes, whereas the lower scatter plot shows the negative correlation on occipital electrodes. The correlation on PO8 reached an *r* of −0.93. The topographies in Figure 4 (C) further underline that frontal amplitudes have a positive correlation with the number of errors, whereas occipital electrodes have a negative correlation with the number of errors.

Due to the high number of comparisons performed in the approach shown in Figure 4, we decided also to calculate the correlations of individually calculated peaks on channels Fz, Cz, Pz and Oz. The results are shown in Table 3. Performance correlated strongest with the amplitude of the N2 component at Oz (*r*  =  −0.86).

**Table 3:** Mean amplitudes, latencies and correlations thereof with BCI performance are shown for N1 (100 and 200 ms),P2 (200 and 250 ms), N2 (250 and 375 ms) and P300 (250 and 700 ms).

	Amplitude vs. performance		Latency vs. performance	
	Mean (*μ*V)	R	Mean (ms)	R
	N1 (100–200 ms)			
Fz	−1.74±1.0	0.54(p = 0.09)	162.29±27.9	–
Cz	−1.42±0.9	–	159.45±25.8	–
Pz	0.32±0.5	−0.59(p = 0.06)	167.61±42.2	–
Oz	**1.75±1.2**	**−0.74** **(p = 0.01)**	163.71±30.0	–
	P2 (200–250 ms)			
Fz	**0.99±2.0**	**0.65** **(p = 0.04)**	235.09±22.8	–
Cz	1.30±1.3	–	236.51±18.3	–
Pz	**−0.25±1.1**	**−0.62 (p = 0.05)**	222.30±22.3	0.57 (p = 0.07)
Oz	**−0.91±1.8**	**−0.77 (p = 0.01)**	233.66±22.9	–
	N2 (250–375 ms)			
Fz	−0.72±1.8	0.57 (p = 0.07)	**315.34±38.0**	**0.71 (p = 0.01)**
Cz	**−0.56±1.1**	**0.71** **(p = 0.02)**	304.69±36.6	–
Pz	0.48±1.4	–	309.30±44.5	–
Oz	**0.42±1.7**	**−0.85 (p = 0.00)**	309.66±28.8	–
	P3 (250–700 ms)			
Fz	1.82±1.9	–	343.04±72.4	–
Cz	2.08±1.3	–	361.51±87.1	–
Pz	−0.74±1.4	–	**424.01±185.5**	**−0.64 (p = 0.03)**
Oz	−2.20±1.4	–	369.67±74.8	–

All correlations were calculated for channels Fz, Cz, Pz and Oz. To improve readability of the table we removed correlations with *p* > 0.1. Furthermore, all correlations with *p* < 0.05 were printed in boldface.

## Discussion

Eleven persons with motor impairments participated in the study using a visual P300 BCI for control of a web browser. It was possible to use features extracted from EEG data recorded during an auditory oddball task for prediction of aptitude. A correlation analysis of the different ERP components showed a strong relationship between early positive and negative potentials around 200 ms with performance. We found this relationship between individual sample points of the whole spatio-temporal feature matrix (up to *r*  =  0.93) and also between individual peaks (up to *r*  =  −0.86).

### BCI Performance

We defined performance as the number of errors needed to complete a task consisting of 40 selections in a P300 7×7 matrix with two 5×6 submatrices. On average the participants made 15.1 errors until performing all 40 selections correctly. The number of errors did not correlate with the degree of impairment. This is in concordance with other studies that did not show a relationship between the ability to use a BCI and disease progression [24]. For better comparability of the performance of the participants in this study with the performance of other samples of persons with motor impairment, the accuracy was estimated as 40 correct selections out of 55.1 total selections. This results in 73% online accuracy which is above the criterion level of 70% accuracy [31], [39]. We believe that the accuracy achieved by the participants in this study can be compared to the accuracy achieved by ALS or persons with other motor disabilities in previous P300 BCI studies [26], [30], [52], [63], [64], [65]. In fact none of the other studies demanded such a complex task from the participants as performed by the participants in this study. Therefore, these results reflect a robust estimation of the ability to control a BCI. Participants needed to make selections using a 7×7 letter matrix with two 5×6 sub-matrices. Additionally, the participants controlled a web browser on an additional screen (requiring attentional switches between the P300 matrix and the screen displaying the web browser) which changed the displayed content accordingly. This increased the demand on attentional resources of the participants. It is also the largest sample of participants to have participated in any of the aforementioned studies. Using novel stimulation techniques such as famous faces instead of flashing rows and columns information transfer rate (ITR) can be increased substantially than what we report in this study [66].

### Comparison of ERPs

ERP differences between healthy participants and participants with severe motor impairment have been explored in several studies. The general conclusion is that besides motor impairment there appears to be an effect of the disease on attention and working memory. In some studies this loss of cognitive funcitons has been linked to the severity of the disease [67]. Other studies found that ALS may be, but must not be linked to frontotemporal dementia (FTD). In samples of persons with FTD the occurence of ALS is much higher than can be expected in a random sample [68]. This has been attributed to the observation that the gene defective in familial ALS is sometimes linked to the gene causing FTD [69], [70]. Thus, the two diseases occur more frequently in the same person than can be expected by chance alone. A summary of cognitive effects associated with ALS can be found in Raaphorst et al. [71]. This is reflected in electrophysiological studies. In an auditory selective attention task with eight persons with ALS, ERPs were reduced compared to age-matched healthy controls [72], [73]. It was also shown that with the progression of impairment auditory and visual P300 latency was increased [74]. Cognitive impairments and decreased P3a/P3b amplitudes as well as higher P3a latencies were also shown [75], [76]. An important factor that may contribute to ERP abnormalities in persons with ALS are periodic failures in the ventilation system that may lead to anoxia [77]. Analysis of intensive care unit patients, especially those receiving artificial ventilation, have revealed significant impairment of cognitive functions [78]. Recent data suggests that abnormalities tend to increase, e.g. in P300 latency, with the disease duration [79], [80]. Compared a sample of healthy participants [44], amplitudes were decreased and latencies increased in the sample of persons with motor impairments in this study.

We found that the minimum amplitude on Cz between 250 and 375 ms indicates fewer errors in the internet browsing task performed by the participants of this study. On Pz the polarity switches (due to the CAR) and increased amplitudes indicate fewer errors. The N2 has been described as more than a sensory component and its involvement in cognitive control processes such as response inhibition has been underlined [81]. This indicates a role of selective attention and arousal in BCI tasks when the participants ignore non-targets and respond to the target stimuli which is reflected in variations in N2 amplitude. We also found that the latency increases with the number of errors on frontal EEG channels.

Amplitude and latency of the P2 have a weaker correlation than amplitude and latency of the N2 [82]. Indeed, we did not find correlations between latency and performance. There are no P2 amplitude differences between targets and non-targets for the low aptitude users. In particular, on channel Cz there was no difference between the P2 elicited by targets and non-targets whereas the averaged target and non-target curves of the high aptitude users diverge after the N1. Our data shows a lower P2 amplitude on frontal channels and higher amplitude on occipital channels for high aptitude users. On all channels the absolute values of the P2 amplitude were higher for low aptitude users.

In fact, the P2 amplitude appears to decrease with increased attention (for a review of the P2 see Crowley and Colrain [83]). An enhancement of the P2 amplitude to unattended stimuli was found by using EEG and MEG [84], [85]. In both studies this attenuation of the P2 in the attend condition coincided with an enhancement of the N1 (which has higher absolute amplitudes in the high aptitude group in this study). The authors explained this with two different stages of selective attention. The first being inhibition, which is visible as the increased N1, and the second being filtering, which is accompanied by a decreased P2 in the EEG. Under this assumption high aptitude users exhibit increased attention and better filtering mechanisms in the target condition compared to the non-target condition and also compared to low aptitude users.

## Conclusions

A short auditory oddball experiment can be used to predict performance in a sample of persons with severe motor impairment. This was shown previously in a sample of healthy participants and also with a person in CLIS [16], [44]. It is probable that a single variable that predicts performance will not be found, due to the many factors that influence performance. As proposed by Kübler [45], these factors include physiological, anatomical and psychological variables (besides technical variables which are beyond the scope of this paper). Physiological variables such as heart rate variability (HRV) [43], the amplitude of the SMR-peak [35], amplitudes of the ERPs used to control the BCI [42] and the volume of the brain-areas recruited during motor imagery in particular in pre- and supplementary motor areas [36] influence performance. Additionally the anatomy of a BCI user may be affected by lesions or neurodegeneration [72] which influences the EEG and consequently BCI performance. Even in healthy users differences in brain-connectivity can predict variations in BCI performance [37]. Finally, psychological factors such as mood and motivation Kleih:2010fk and visuo-motor coordination [33] have an added impact on the ability to control a BCI. The factors which we found in this study are clearly physiological of origin but are probably a measure of a psychological variable: increased attention and better filtering mechanisms (which seem to be persistent throughout a BCI session). Eventually, all predictors should be combined and redundancies removed. Clearly some predictors cannot be applied in a feasible manner with every BCI user, e.g. the predictors requiring magnetic resonance imaging (MRI) measurements. Nonetheless, they provide valuable input to the overall model and point to negative influences on BCI performance that may be removed without the need for continuous MRI measurements.

In combination with future investigations of BCI performance these methods can be used to select the optimal paradigm for persons with motor impairments [86]. Additional improvements will be possible using EEG features to monitor BCI performance during usage. Findings as in this paper may be used as a control variable to specifically train the variables in question (such as attention). This will increase the chance that early BCI sessions are successful and not frustrating experiences.
